# Endocrine disruption of vitamin D activity by perfluoro-octanoic acid (PFOA)

**DOI:** 10.1038/s41598-020-74026-8

**Published:** 2020-10-08

**Authors:** Andrea Di Nisio, Maria Santa Rocca, Luca De Toni, Iva Sabovic, Diego Guidolin, Stefano Dall’Acqua, Laura Acquasaliente, Vincenzo De Filippis, Mario Plebani, Carlo Foresta

**Affiliations:** 1 Department of Medicine, Unit of Andrology and Reproductive Medicine, University of Padova, Via Giustiniani, 2, 35128 Padova, Italy; 2 Department of Neurosciences, University of Padova, via Gabelli 65, 35128 Padova, Italy; 3 Department of Pharmaceutical Science, University of Padova, via Marzolo 5, 35128 Padova, Italy; 4 Department of Pharmaceutical and Pharmacological Sciences, University of Padova, via Marzolo 5, 35128 Padova, Italy; 5 Department of Medicine, Laboratory Medicine, University of Padova, Via Giustiniani, 2, 35128 Padova, Italy; 6 Fondazione Foresta ONLUS, via Gattamelata 11, 35128 Padova, Italy

**Keywords:** Metabolic bone disease, Translational research

## Abstract

Perfluoroalkyl substances (PFAS) are a class of compounds used in industry and consumer products. Perfluorooctanoic acid (PFOA) is the predominant form in human samples and has been shown to induce severe health consequences, such as neonatal mortality, neurotoxicity, and immunotoxicity. Toxicological studies indicate that PFAS accumulate in bone tissues and cause altered bone development. Epidemiological studies have reported an inverse relationship between PFAS and bone health, however the associated mechanisms are still unexplored. Here, we present computational, in silico and in vitro evidence supporting the interference of PFOA on vitamin D (VD). First, PFOA competes with calcitriol on the same binding site of the VD receptor, leading to an alteration of the structural flexibility and a 10% reduction by surface plasmon resonance analysis. Second, this interference leads to an altered response of VD-responsive genes in two cellular targets of this hormone, osteoblasts and epithelial cells of the colorectal tract. Third, mineralization in human osteoblasts is reduced upon coincubation of PFOA with VD. Finally, in a small cohort of young healthy men, PTH levels were higher in the exposed group, but VD levels were comparable. Altogether these results provide the first evidence of endocrine disruption by PFOA on VD pathway by competition on its receptor and subsequent inhibition of VD-responsive genes in target cells.

## Introduction

Perfluoroalkyl substances (PFAS) are a family of artificial molecules characterized by fluorinated hydrocarbon chains^1^. Because of their unique chemical and physical properties, ranging from oil and water repellence to temperature and chemical resistance, PFAS are used in a wide range of consumer products and industrial applications, including oil and water repellents, coatings for cookware, carpets and textiles. Their attractive physiochemical characteristics also drive persistent accumulation into the environment, making them a potential biohazard for human health, acting as endocrine disrupting chemicals (EDCs)^2^. This adds to the total burden of chemicals to which people are exposed and increases the risk of health impacts. Of the relatively few well-studied PFAS, most are considered moderately to highly toxic, particularly for children’s development. Perfluoro-octanoic acid (PFOA) is one of the predominant form in human samples and has been shown to induce severe health consequences, such as neonatal mortality, neurotoxicity, and immunotoxicity^3^.

Epidemiological studies have reported a potential involvement of PFAS in metabolic alterations, such as glucose homeostasis, metabolic syndrome, body weight, and insulin resistance^4–7^. In addition, recent studies have also shown the positive associations between serum levels of PFOA and PFOS with altered lipid profiles, including total cholesterol, low-density lipoprotein (LDL)-cholesterol, high-density lipoprotein (HDL)-cholesterol, and triglycerides^8^. Interestingly, adipocyte differentiation was favored by PFAS exposure in fibroblasts^9^, suggesting a role in the perturbation of the precursor lineage of both bone and fat cells.

Toxicological studies indicate that PFAS accumulate in bone tissues and could cause altered bone development^10^. Furthermore, experimental and human autopsy evidence suggests accumulation of PFAS in the skeleton^11–13^. Epidemiological studies have reported an inverse relationship between PFAS exposure and bone health^14–19^, suggesting a possible role of PFAS in the onset of osteoporosis, however the associated mechanisms are still unexplored. Because different PFASs, including PFOA, have been associated with altered sex steroid^20,21^ and thyroid hormone levels^22–24^ we hypothesized that they may also affect vitamin D metabolism. Vitamin D (VD) is a key regulator of bone mineralization and calcium homeostasis in both men and women^25^, and low levels of VD are typically associated with reduced bone mass. In order to be biologically active, vitamin D must be converted to its active form, 1,25-dihydroxyvitamin D (1,25(OH)D), by two sequential hydroxylation steps catalysed by 25-hydroxylase and 1α-hydroxylase. In the presence of VD insufficiency or deficiency, intestinal calcium absorption is reduced below optimal levels and there is a compensatory feedback to parathyroid glands that leads to an increase of PTH levels, a condition known as secondary hyperparathyroidism, with a subsequent stimulation of bone resorption to increase circulating calcium levels, ultimately leading to accelerated bone loss^26^. VD levels can be influenced by a variety of known factors, such as sun exposure, diet, age, and obesity^27^. In addition, VD homeostasis may also be influenced by EDCs because the molecular structure of the active metabolite of VD, 1,25(OH)D, is very similar to classic steroid hormones, being cholesterol their common precursor, and VD receptor (VDR) is similar to steroid and thyroid hormone receptors. Acting as an endocrine disruptor, we hypothesize a possible involvement of PFOA on vitamin D metabolism, as already reported for other chemicals. Two studies on adult NHANES participants and pregnant women reported inverse associations of bisphenol A and phthalates with total 25-hydroxyvitamin D (25(OH)D) levels, with some differences in associations by gender and race/ethnicity^28,29^. While PFAS are also EDCs that may affect estrogen^30,31^, androgen^21^ or glucocorticoid^32,33^ activity, previous studies have examined whether PFAS exposures are associated with vitamin D levels. Association of two PFAS with total serum 25(OH)D levels was found in a nationally representative sample of the United States population^34^. Higher serum PFOS concentrations were also associated with increased odds of being vitamin D deficient. Limited laboratory research suggests that EDCs may affect vitamin D homeostasis, however the mechanism is still unknown.

To this end, we evaluated the possible mechanisms of PFAS interference on vitamin D action. In particular, computational and in vitro approaches aimed to study the interaction of PFOA on the binding of VD on its receptor and the consequent functional activity in target cells, such as osteoblasts and intestinal cells. Finally, hormonal profile was evaluated in a sample of young men exposed to PFAS contamination, compared with age-matched controls.

## Results

**Figure 1:**
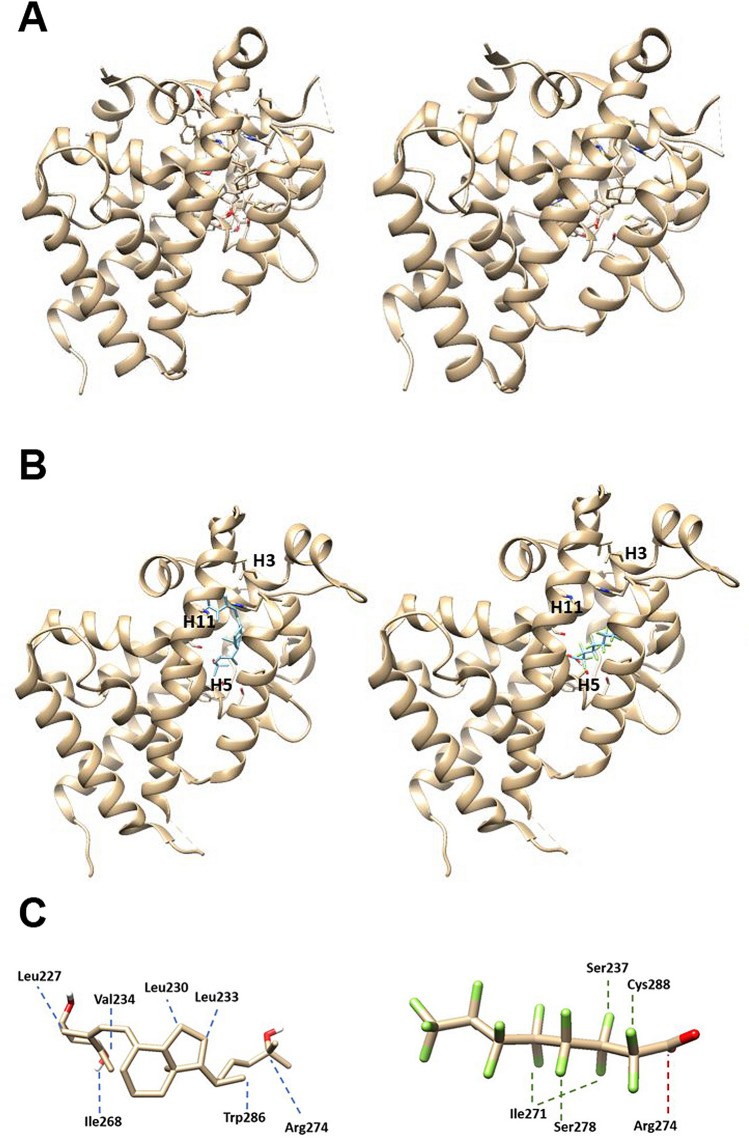
(**A**) *Danio rerio* VDR bound to 1,25(OH)D: experimental complex is shown on the left panel; on the right the complex obtained by using the computational docking procedure. (**B**) Best docking solutions for the molecular complex between human VDR (from PDB model 1IE8) and 1,25(OH)D (left) or PFOA (right). Both molecules were predicted to share the same binding domain corresponding to the known ligand-binding pocket of the receptor (see methods for details). (**C**) Details of the estimated binding mode of 1,25(OH)D (left) and PFOA (right). As expected, estimated 1,25(OH)D binding involved a network of hydrogen and hydrophobic bonds (blue lines) mainly with residues in the 227–278 region. PFOA binding was predicted to occur in the same region of the receptor and appeared characterized by a network of halogen bonds (green lines). A possible salt bridge (red line) involving Arg274 was also identified. Docking of ligands to the receptor were performed by using the *AutoDock Vina* software v.1.1.2. (https://vina.scripps.edu/).

In order to investigate whether PFOA interferes with the binding of 1,25(OH)D on its receptor, as observed also for other steroid receptors, we first computationally assessed the molecular competition of the two agonists on the VDR by molecular docking analysis. To test the accuracy of the aforementioned computational procedures, the docking of 1,25(OH)D model was first tested to the *Danio rerio* VDR structure, in order to compare the result of the computational approach to the available experimental model of the complex^35^. The obtained docking pose of the calcitriol molecular model in the *Danio rerio* VDR was consistent with the experimental finding (Fig. 1A), indicating that the applied computational procedure was able to correctly identify the binding site. When docking between human VDR and calcitriol or PFOA was performed, both molecules docked in the same receptor site (Fig. 1B), located among helices 3, 5 and 11. As schematically illustrated in Fig. 1C, the predicted binding of PFOA was mainly through a network of halogen bonds involving Ser237, Ile271, Ser278 and Cys288. The best energy scores (ΔG) associated with the binding of 1,25(OH)D and PFOA resulted of − 12.1 kcal/mol and − 9.0 kcal/mol respectively, suggesting a lower estimated affinity of PFOA for the binding site.

**Figure 2:**
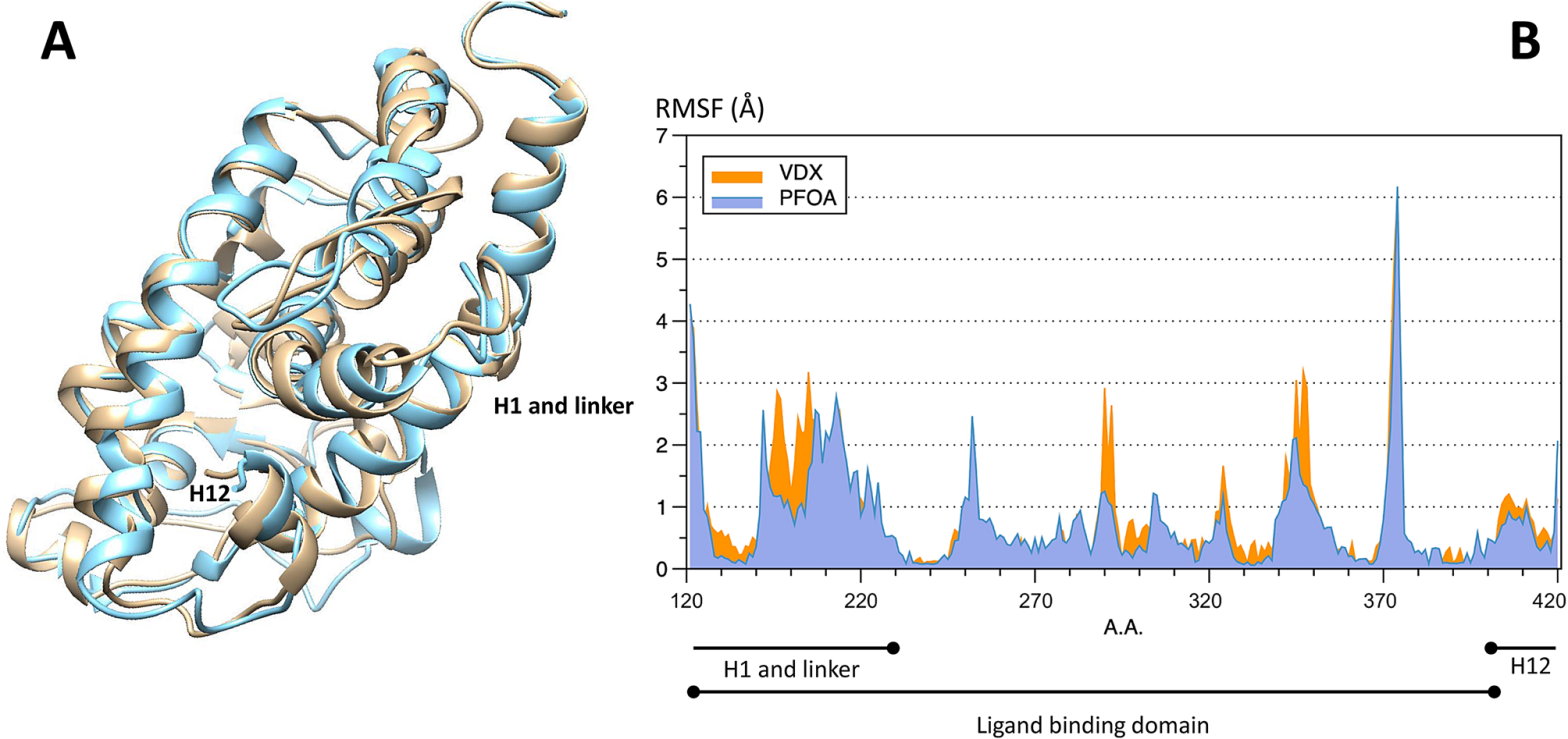
(**A**) Aligned representative configurations from the estimated set of configurations the VDR protein may exhibit when in complex with calcitriol (yellow) and PFOA (cyan). (**B**) Protein flexibility of human VDR in the two cases as estimated by the applied molecular dynamics approach (see methods for details). As illustrated, major differences in flexibility were estimated to occur not only in the ligand binding domain, but also in the hinge and in the C-terminal (H12) regions. These estimated configurational changes may influence the interaction of VDR with co-activator molecules and the final signal transduction. Figures were obtained with Cabs-Flex 2.0 online free-to-use software. Available at: https://biocomp.chem.uw.edu.pl/CABSflex2.

**Figure 3:**
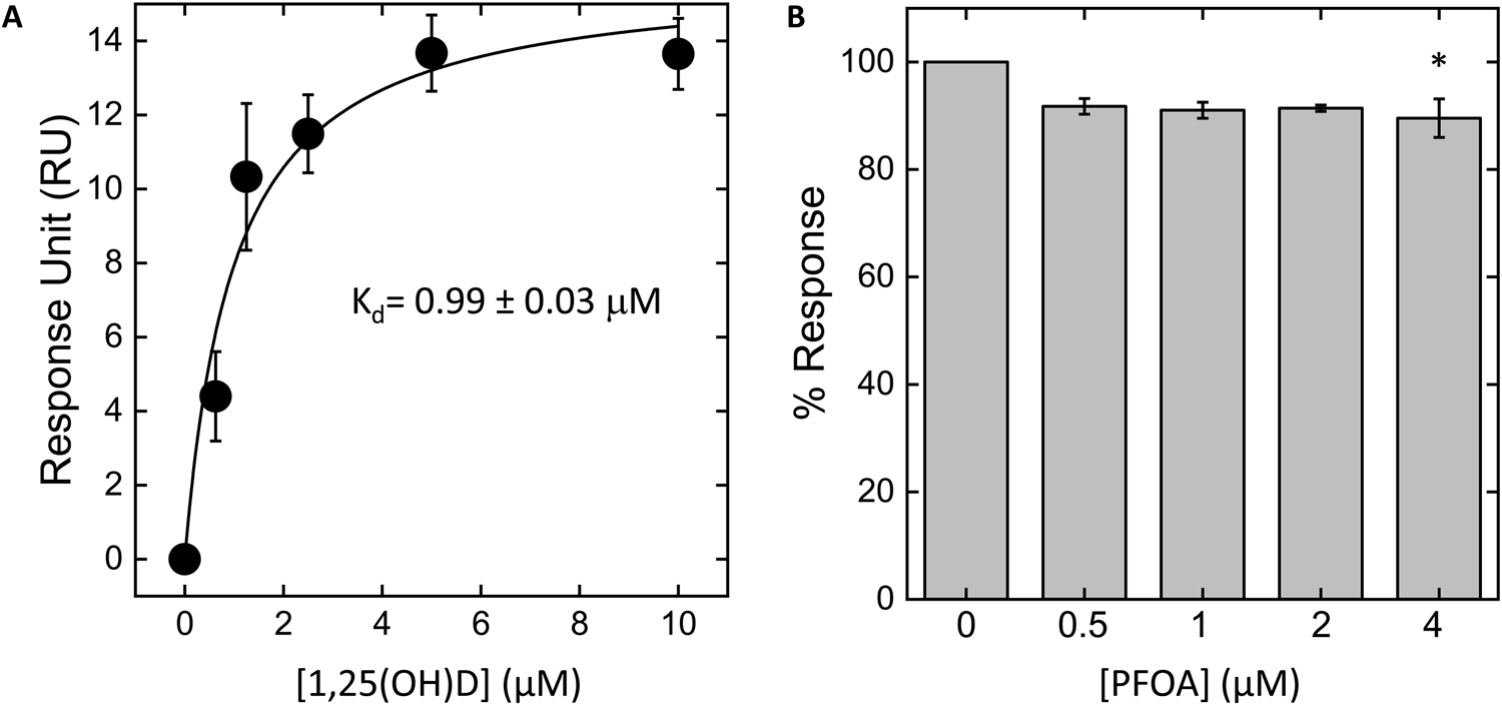
Binding of 1,25(OH)D to immobilized Vitamin D receptor is reduced by PFOA. (**A**) Solutions of 1,25(OH)D were injected at a flow rate of 10 µL/min at 25 °C, using PBS containing 5% DMSO (v/v) as running buffer. Each SPR trace was subtracted for unspecific binding (i.e.,2% of RU_max_). The response units (RU) at the steady state were plotted as a function of [1,25(OH)D] and fitted to the Langmuir equation to yield the dissociation constant Kd. (**B**) Competition experiment in the presence of PFOA. A 10 µM solution of 1,25(OH)D was incubated with different concentrations of PFOA (0 to 4 µM) for at least 15 min before injection over the same Vitamin D-coated sensor chip. Results are shown as the maximal association RU (expressed as the percentage relative to the response measured without PFOA) achieved at increasing concentrations of PFOA. *p < 0.05 vs. basal.

As shown in Fig. 2, the applied molecular dynamics approach indicated that the binding of PFOA to VDR can lead to a receptor protein configuration set characterized by a different profile of chain flexibility when compared with the 1,25(OH)D-bound VDR configuration. Interestingly, in addition to the binding domain, major changes in flexibility were estimated to occur in the hinge region of the receptor and in the C-terminal transactivation domain, where overall residues showed lower fluctuations in PFOA-bound than in 1,25(OH)D-bound receptor. SPR measurements were performed to monitor the real-time interaction between 1,25(OH)D and PFOA with the VDR. In this experiment, the VDR was immobilized on a CM5 sensor chip, and solutions of 1,25(OH)D (0–10 µM) and PFOA (0–4 µM) were injected separately at different concentrations. Despite the low molecular weight of 1,25(OH)D (401 Da), SPR resolved the interaction and provided a dissociation constant Kd = 0.99 ± 0.03 µM (Fig. 3A). Next, we performed a competition experiment to assess whether the presence of PFOA would reduce the binding of 1,25(OH)D to VDR. We incubated a solution of 1,25(OH)D with different concentrations of PFOA, and the resulting mixture was flowed over the same VDR-coated sensor chip. At the highest concentration tested, we observed a small but significant 10% decrease of 1,25(OH)D binding, suggesting that the presence of PFOA reduces the binding of 1,25(OH)D to its receptor (Fig. 3B). Higher concentrations of PFOA were not tested because of the limited solubility in the running buffer. Smaller concentrations of 1,25(OH)D were not tested because of the instrument sensitivity.

**Figure 4:**
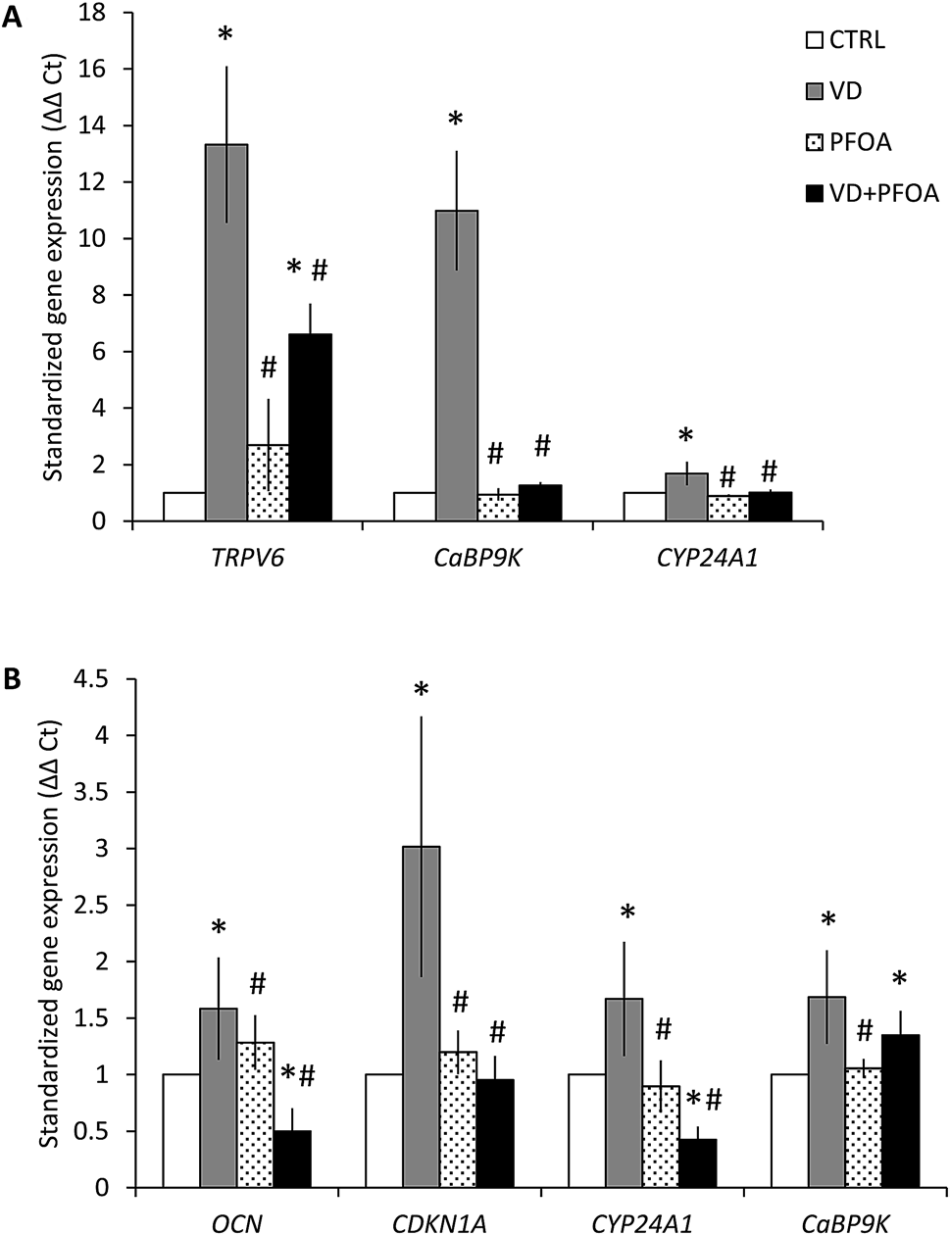
(**A**) Quantitative RT-PCR on human Caco-2 cells stimulated with Vitamin D and PFOA. Relative gene expression of Transient Receptor Potential Cation Channel Subfamily V Member (*TRPV6*), Calbindin-D9k (*CaBP9K*) and Cytochrome P450 Family 24 Subfamily A Member 1 (*CYP24A1*) after 24 h stimulation with 1,25-dihydroxyvitamin D (VD, grey bars), PFOA (dotted bars) or both (VD + PFOA, black bars), normalized to the expression of the housekeeping GAPDH on control (CTRL, no stimulation, white bars). Data are shown as the mean ± standard deviation (SD) of three different experiments performed in triplicate. (**B**) Quantitative RT-PCR on human SAOS-2 cells stimulated with Vitamin D and PFOA. Relative gene expression of osteocalcin (*OCN*), p21 (*CDKN1A*), Cytochrome P450 Family 24 Subfamily A Member 1 (*CYP24A1*) and Calbindin-D9k (*CaBP9K*) after 24 h stimulation with 1,25-dihydroxyvitamin D (VD, grey bars), PFOA (dotted bars) or both (VD + PFOA, black bars), normalized to the expression of the housekeeping GAPDH on control (CTRL, no stimulation, white bars). Data are shown as the mean ± standard deviation (SD) of three different experiments performed in triplicate. *p < 0.05 vs. CTRL; #*p * <  0.05 vs. VD.

**Figure 5:**
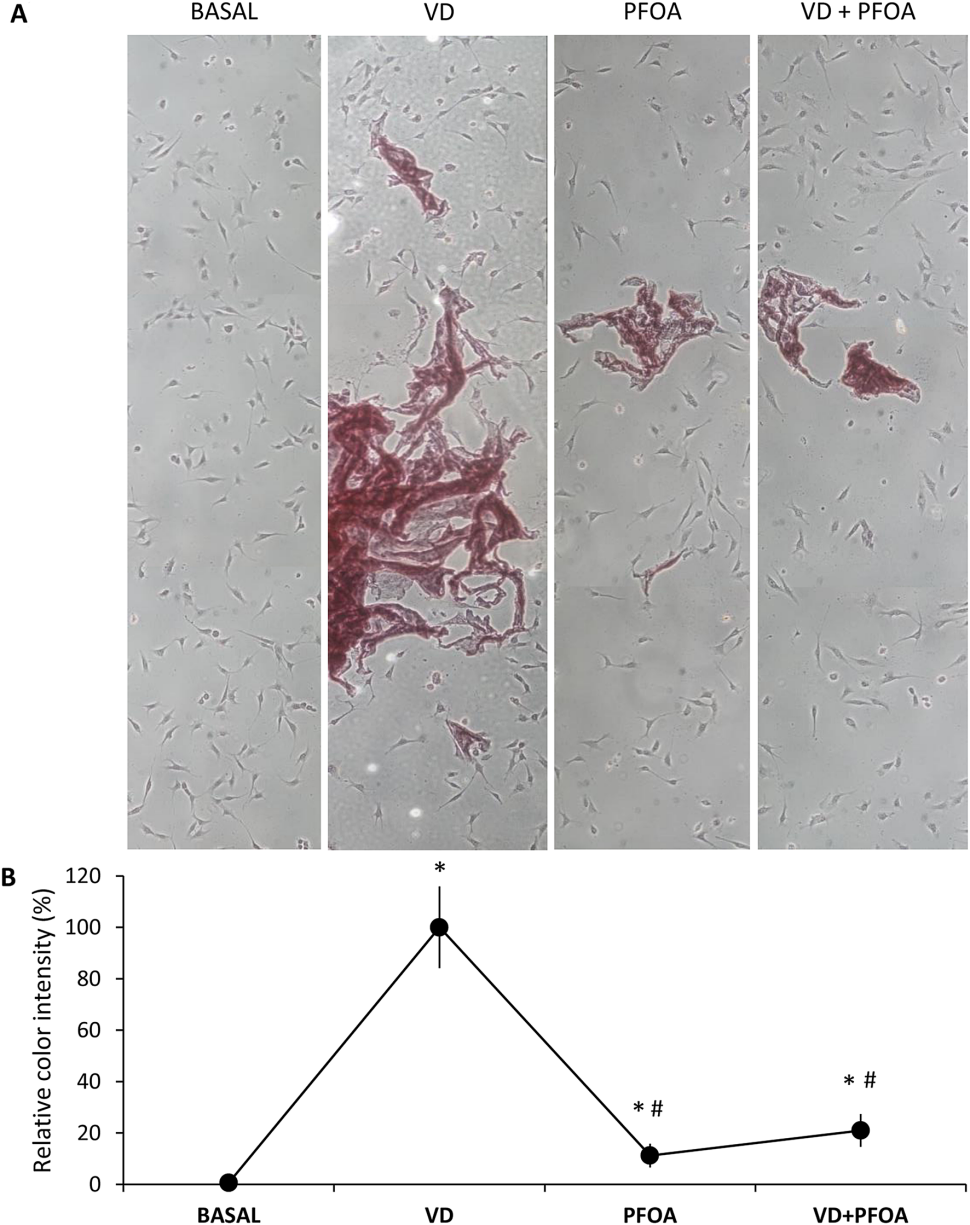
Mineralizing ability of human SAOS-2 cells stimulated with Vitamin D and PFOA. (**A**) Representative images of mineralized nodule formation detected by Alizarin red-S staining in the presence of PFOA (400 ng/mL) compared to that of 1,25(OH)D (VD, 100 nM) used as positive control. Each image is the result of 4 different pictures at 10X magnification merged together. (**B**) Relative intensity of red was calculated by computer assisted image analysis with ImageJ software. Mean red intensity was calculated from three replicates within each experimental condition. Data was standardized to the positive control (VD) and expressed as fold intensity (%) ± SD. *p < 0.05 vs. basal; #*p * < 0.05 vs. VD.

To assess whether the reported interference of PFOA on VDR by computational models could also translate into functional alterations of 1,25(OH)D action in cellular systems, we analysed the expression of important target genes of 1,25(OH)D in human Caco-2 and SAOS-2 cell lines, based on previous studies^36–39^. In human epithelial colorectal adenocarcinoma cells we observed a significant upregulation of genes encoding TRPV6 channel, Calbindin-D9k and CYP24A1 upon stimulation with 1,25(OH)D 100 nM for 24 h, which was blunted upon co-incubation with 400 ng/mL PFOA, whereas incubation with PFOA alone did not alter gene expression compared with controls (Fig. 4A). OCN and CDKN1A were below detection limit. In human osteosarcoma cells, after 24 h stimulation with 1,25(OH)D 100 nM we observed a significant upregulation of *OCN*, *CDKN1A, CaBP9K* and *CYP24A1*, which was blunted upon co-incubation with PFOA, with the exception of CaBP-9 K. PFOA alone did not elicit significant modifications of gene expression (Fig. 4B). In order to understand whether the above interference of PFOA on 1,25(OH)D target genes in osteoblasts has an impact on cell physiology, we assessed bone mineralization by Alizarin red (Fig. 5A). We observed a massive mineralization after 15 days of 1,25(OH)D stimulation, which was significantly reduced upon co-incubation with PFOA (Fig. 5B). PFOA alone induced a slight increase in mineralization compared with unstimulated controls, but much lower compared with 1,25(OH)D. Cell viability or number was not altered in cells incubated with PFOA compared with 1,25(OH)D (data not shown). Next, we evaluated the biochemical and hormonal profile of a small sample of young men (n = 100) with high or low exposure to PFOA. By LC–MS we confirmed higher PFOA serum levels in the group of subjects with known geographical exposure to PFAS (n = 50), compared with age-matched control subjects (n = 50; PFOA = 4.14 ± 1.68 and 14.02 ± 1.35, respectively; *p* < 0.001). Table 1 shows subjects characteristics: the two groups did not differ in age, BMI, alcohol consumption or smoking. Calcium intake did not differ between groups (data not shown). Hormonal analyses shows comparable levels of serum 25(OH)D between groups, but serum PTH was almost twice in exposed subjects compared with controls (Table 1). Finally, serum calcium and phosphate did not differ between groups.

**Table 1:** Characteristics of the study population stratified by PFAS exposure.

	Controls (N = 50)	Exposed (N = 50)	*p* value^a^
Age (years)	18.9 ± 1.1	19.2 ± 1.2	0.417
BMI	22.8 ± 2.1	22.7 ± 2.5	0.978
Smoking (%)	25%	30%	0.616
Alcohol consumption (%)	12,5%	10%	0.723
25OH-D (nmol/L)	54.9 ± 20.1	62.8 ± 14.6	0.163
PTH (ng/dL)	4.9 ± 2.1	8.4 ± 3.8	**0.001**
Calcium (mM)	2.48 ± 0.08	2.53 ± 0.08	0.051
Phosphate (mM)	1.49 ± 0.40	1.55 ± 0.35	0.590
Serum PFOA (ng/mL)	4.14 ± 1.68	14.02 ± 1.35	** < 0.001**

^a^Chi-square test (nominal data) or Mann–Whitney U test (continuous data) was performed.

Significant p values are in bold.

## Discussion

In this study we report different degrees of evidence pointing towards the endocrine interference of PFOA on vitamin D activity in target cells. Since VD is reported to regulate osteoblast proliferation and differentiation, bone mineralization and gene transcription of importance for the bone and calcium homeaostasis^40–42^, our results showing PFOA inhibition of VD-induced gene expression and reduced bone mineralization would provide the first biological explanation of the epidemiological reports indicating PFAS endocrine disruption on bone health. Together with computational and in silico evidence indicating a competition of PFOA on the same binding site of 1,25(OH)D on the VDR, and subsequent alteration of receptors flexibility, this study adds further knowledge to the range of steroid pathways disrupted by PFAS^20–24^. However, in a small cohort of young healthy men from PFAS-exposed community, we did not observed a reduction of 25(OH)D levels, despite a raise in PTH levels that could suggest a compensatory mechanism by parathyroid glands to maintain calcium homeostasis in front of a functional defect of VD action.

In the experimental setting to investigate bone metabolism, we used osteosarcoma Saos-2 cells as a model for osteoblast-like cells. This cell line has been used in many previous studies as a model for osteoblasts, and express VDR and VD metabolic enzymes^40^. Among these enzymes, CYP24A1 is induced by 1,25(OH)D in target tissues to inactivate 1,25(OH)D in order to avoid excessive action and prevent toxicity^43^. Inhibitory effects on this enzyme should therefore lead to an increased effect of vitamin D in the cell, which may have a strong impact on cellular function, as demonstrated for glucocorticoids, with consequent alteration of bone metabolism^40^. The observed dysregulation of this enzyme by PFOA in human osteoblasts could have a negative effect on bone. 1,25(OH)D initiates many responses in osteoblasts as well as other cell types: in osteoblasts, 1,25(OH)D have been shown to inhibit cell cycle progression at the G1 phase and to promote cell differentiation^44^, and this involves p21 gene upregulation^45^. It has been previously demonstrated that the p21 gene promoter region includes VDR binding sites^46^, and that p21 gene expression is triggered by multiple differentiation inducing agents including 1,25(OH)D^38^. Increased p21 expression therefore causes cell cycle arrest and suppression of cell growth in response to 1,25(OH)D stimulation. Inhibition of osteoblast proliferation by 1,25(OH)D can signal their differentiation and induce the up-regulation of the genes that are expressed only during their progression to the mineralization phase. *OCN* is one such gene that has been shown to be induced by 1,25(OH)D in mature osteoblasts^47^. The 5′-flanking region for the gene for *OCN* has a characterized VD responsive element which infers the positive regulation by the ligand-bound VDR. The lack of p21 upregulation by 1,25(OH)D upon PFOA co-incubation therefore could lead to lack of differentiation and induction of mineralization, as outlined by decreased OCN response under the same experimental conditions. OCN is a marker for osteoblastic differentiation and an important indicator for matrix maturation and mineralization and 1,25(OH)D helps promote differentiation toward a functionally mature osteoblastic phenotype as suggested by increased *OCN* expression and mineralization. In order to fully understand how the decreased inactivation of 1,25(OH)D might translate in osteoblast function, we analyzed cell mineralization by Alazirin red. As expected, 1,25(OH)D was a potent stimulator of mineralization, but the addition of PFOA significantly blunted the presence of calcium deposits. It might be speculated that increased levels of 1,25(OH)D due to suppression of its inactivation could lead to unbalanced VD homeostasis within cells, thereby leading to inhibition of osteoblast maturation, finally leading to altered bone mineralization. Overall, the 10% reduction in 1,25(OH)D binding to VDR in the presence of PFOA could explain the observed biological effects, although the binding competition is relatively small, it should kept in mind that due to instrument sensitivity the tested concentrations of 1,25(OH)D were an order of magnitude higher than in physiological conditions, but comparable PFOA levels could not be tested due to its low solubility. Therefore, the ratio between 1,25(OH)D and PFOA is unbalanced, and in the presence of lower 1,25(OH)D concentrations the reduced binding to VDR could even more pronounced in the presence of PFOA.

VD also increases calcium levels by enhancing uptake of calcium via the intestine, which is mediated by expression of several calcium transport genes including the transient receptor potential cation channel, subfamily V, member 6 (TRPV6) and calbindin-D9k (CaBP-9 k)^48^. TRPV6 is a membrane calcium channel responsible for the first step of calcium absorption in the intestine. CaBP-9 k facilitates the transport of calcium across the enterocytes from the apical region of the intestine^49^. Both TRPV6 and calbindin-D9k are major targets of 1,25(OH)D associated with calcium homeostasis^50,51^. In addition to VD, estrogens are potential regulators of genes associated with calcium regulation and are involved in intestinal calcium absorption, therefore estrogenic EDCs could also modulate these genes. Among EDCs, Bisphenol A reduced the expression of both TRPV6 and CaBP-9 k in the duodenum^52^. Altogether these results provide a novel explanation to support the observed alteration of bone metabolism in PFOA-exposed animals and subjects, however, our in vitro experimental approach needs to be confirmed in vivo*.*

Our findings suggest that PFOA has negative effects on the expression of genes involved in calcium transfer, which would disturb calcium absorption in the intestine, resulting in a negative feedback on the parathyroid glands. Normally, in the presence of inadequate vitamin D status, calcium absorption is lower than optimal and there is a compensatory increase in PTH levels (secondary hyperparathyroidism), with a subsequent stimulation of bone reabsorption and accelerated bone loss. However, in the presence of normal VD levels but reduced activity due to endocrine-disrupting properties of PFOA in target cells, we could hypothesize a functional hypovitaminosis D, due to reduced activity of 1,25(OH)D and driven by lower intestinal calcium absorption, which is masked by normal serum 25(OH)D levels and ultimately leads to increased PTH as a compensatory mechanism. The few available data is not in agreement on the impact of PFAS exposure on VD levels^16,34^, possibly due to different settings, serum PFAS distributions and populations. In our cohort, it could be possible that the young age of subjects and the lack of known risk factors (obesity, alcohol, use of drugs) could compensate the inhibitory effect of PFOA in reducing circulating VD levels. However, our experimental results would support rather a functional hypovitaminosis D, as unmasked by compensatory secondary hyperparathyroidism. Our findings in a relatively small sample of exposed subjects and age-matched controls revealed comparable VD levels between groups, but increased PTH in exposed group, although serum calcium and phosphate were not altered. If PFOA does interfere with vitamin D metabolism, it could lead to impaired skeletal development and osteoporosis. Further investigations regarding mechanisms underlying the effects of PFOA on skeletal defects will be required.

There are several study limitations, prominent being a small sample size and lack of generalizability as all our participants were young healthy males. Additionally, due to our cross-sectional study design, we cannot conclude causality of any relationships. Nevertheless, to our knowledge, this is the first study to report an involvement of PFOA on vitamin D activity, by proposing different degrees of evidence in experimental models and human subjects. Despite the convincing biological effect, the mechanism of inhibition remains elusive and requires more biochemical investigations to be unveiled. Based on these results and the available epidemiological data, PFAS-exposed communities would benefit from the assessment of vitamin D status and consequent VD supplementation to counteract PFOA antagonism on VDR.

## Conclusions

Here, we present computational, in silico and in vitro evidence supporting the interference of PFOA on vitamin D action at different degrees. First, PFOA competes with calcitriol on the same binding site of the vitamin D receptor (VDR), leading to an alteration of the structural flexibility of the receptor. Second, this interference leads to an altered response of vitamin D-responsive genes in two cellular targets of this hormone, osteoblasts and epithelial cells of the colorectal tract. Third, mineralization in human osteoblasts is reduced upon co-incubation of PFOA with calcitriol. Finally, in a cohort of young healthy men, VD was not decreased in the exposed group, but PTH levels were higher in association with PFAS exposure, suggesting a compensatory mechanism in response to functional hypovitaminosis D. Altogether these results provide a possible explanation to skeletal alterations associated with PFAS exposure, and could provide a therapeutic target to overcome endocrine disruption by these chemicals.

## Material and methods

### Docking analysis

The possible association between PFOA molecule and human nuclear receptor for vitamin D (VDR) was theoretically investigated by docking methods. In the field of molecular modelling these methods lead to the prediction of the possible binding sites between two molecules allowing the formation of a stable complex. To perform the analysis, the starting point is usually the tertiary structure of the involved molecules. The models of perfluorooctanoic acid (PFOA, C_8_HF_15_O_2_) and 1,25(OH)D (VDX, C_27_H_44_O_3_) were obtained from the PubChem database (https://pubchem.ncbi.nlm.nih.gov; PubChem CID: 9554 and 5280453 respectively). For what it concerns the human VDR, the model by Tocchini-Valentini et al.^53^, experimentally derived from X-ray crystallography data, was obtained from the Protein Data Bank (https://www.rcsb.org; PDB code: 1IE8). It provides a representation of the tertiary structure of the region 120–423 of the receptor, including the 1,25(OH)D binding domain (227–278) part (residues 120–226) of the hinge region (linking the N-terminal DNA binding domain to the large ligand binding domain that includes the calcitriol binding region^53^) and the 416–423 motif which is a transactivation domain. Chosen structures were prepared for docking according to standard procedures. All extra molecules such as ligands or additional subunits were first removed from the human VDR molecular model. Then, all the molecules of interest (receptor and possible ligands) underwent processing steps including deletion of water molecules, repairing of truncated chains, adding of hydrogens and partial charge assignment. The obtained structures were finally stored for further processing. All these calculations were performed by using the *DockPrep* module available in the *UCSF Chimera* molecular modelling software (Resource for Biocomputing, Visualization, and Informatics, University of California, San Francisco; https://www.rbvi.ucsf.edu/chimera). The molecular structure of VDR was energy minimized by using the Yasara software (https://www.yasara.org/minimizationserver.htm)^54^ and docking of ligands to the receptor was then performed by using the *AutoDock Vina* software^55^. The predicted molecular complexes were scored according to Gibbs free energy of binding and RMSD values and for each studied ligand the docking solution with the best scores was considered. A theoretical estimate of the binding affinity to VDR exhibited by each analyzed ligand was obtained by using the PRODIGY tool (https://bianca.science.uu.nl/prodigy/)^56^.

To test the accuracy of the abovementioned computational procedures, the docking of PubChem 1,25(OH)D model to the *Danio rerio* VDR structure (PDB code: 2HC4) was also performed in order to compare the result of the computational approach to the available experimental model of the complex^35^.

### Molecular dynamics

The obtained receptor-ligand complexes finally underwent a molecular dynamics procedure based on the method by Kuriata et al.^57^, available as a web server (https://biocomp.chem.uw.edu.pl/CABSflex2). This method performs a coarse-grained simulation of near-native dynamics of globular proteins^58^ allowing a structural description of protein flexibility in terms of 3D models.

### Surface plasmon resonance (SPR) analyses

SPR experiments were performed on a BIAcore-X100 instrument (GE-Healthcare, Chicago, IL) to monitor the interaction between PFOA and Vitamin D-receptor. Vitamin D-receptor (VDR) (Abcam, Cambridge, MA) was covalently immobilized on a CM5 sensor chip using an amine-coupling chemistry. Binding experiments were carried out by injecting increasing concentrations of 1α,25-hydroxyvitamin D3 (1,25(OH)D, 0–10 µM; Sigma Aldrich, St. Louis, MO) at a flow rate of 10 µL/min, using PBS containing 5% DMSO (v/v) as running buffer. In each assay, a solvent correction cycle was done before and after the concentration series. Eight different DMSO concentrations in PBS running buffer ranging from 4.5% (v/v) to 5.8% (v/v) were injected. The response units (RU) at the steady state were plotted as a function of [1,25(OH)D], and the dissociation constant (Kd) was obtained as a fitting parameter of a binding isotherm. Competition experiments were performed to investigate the effect of PFOA on 1,25(OH)D-VDR interaction. Solutions of 1,25(OH)D (10 µM) were incubated with different concentrations of PFOA (0–4 µM) for 15 min and then injected over the VDR-coated sensor chip. All experiments were performed in triplicate at 25 °C.

### Cell culture

Human osteosarcoma Saos-2 cells (ATCC HTB-85) were cultured in McCoy’s 5A medium with 1.5 mM of L-glutamine (ATCC) supplemented with 100 U/mL of penicillin, 100 U/mL of streptomycin (Sigma, Saint Louis, MO, USA), and 15% fetal bovine serum (FBS, Gibco-Fisher Sci., Waltham, MA, USA). The cells were grown at 37 °C in atmosphere of 5% CO_2_. For the Alizarin Red Staining cells were incubated for 15 days with 1,25(OH)D (100 nM), PFOA (400 ng/mL, Wellington Laboratories, Southgate, ON, Canada, CAS 335-67-1, purity > 98%), or a combination of both, changing the medium every 3–4 days. Chemicals were dissolved in Methanol to a stock concentration of 0.01 M and stored at − 20 °C until use. For gene expression experiments, cells were incubated for 24 h with VD (100 nM), PFOA (400 ng/mL), or a combination of both.

Epithelial colorectal adenocarcinoma Caco-2 cells (HTB37, ATCC) were cultured in Dulbecco’s Modified Eagle’s Medium (DMEM) + 10% FBS, 10 mM non-essential amino acids, 200 mM Lglutamine, 100 U/L penicillin, 100 µg/L streptomycin mixture, 100 mM sodium pyruvate, 50 mg/mL gentamycin sulfate, and 10 mM HEPES. Cells were incubated at 37 °C, in 5% CO_2_ atmosphere. At confluence, cells were stimulated with 1,25(OH)D (100 nM), PFOA (400 ng/mL), or a combination of both, for 24 h.

### Alizarin Red-S staining

The presence of calcium deposits in cells was detected by staining with Alizarin Red-S (Sigma, Saint Louis, MO, USA) as previously described^59^. Osteoblasts cultured in 6-well plates were washed with PBS and fixed in 4% (v/v) formaldehyde (Sigma–Aldrich) at room temperature for 20 min. Cells were then washed twice with excess deionized water prior to addition of 2% alizarin red-S (pH 4.1) per well. Plates were incubated at room temperature for 30 min with gentle shaking. After aspiration of the unincorporated dye, wells were washed four times with deionized water while shaking for 5 min. Brown/red staining was visualized using an inverted microscope (Nikon), and the representative pictures were photographed. Experiments were performed in triplicate. Computer assisted image analysis of color intensity was performed with ImageJ software.

### RNA isolation, cDNA synthesis and real time PCR

Total RNA was extracted from human osteoblast and epithelial colorectal adenocarcinoma cells by RNeasy Mini Kit (QIAGEN, Valencia, CA, USA). Dnase treatment was performed using Ambion TURBO DNA-free Kit (Thermo Fisher Scientific, Carlsbad, CA, USA) according to the manufacturer's instruction. cDNA synthesis from total RNA (100 ng) was carried out using SuperScript III (Invitrogen, Carlsbad, CA, USA) and random hexamers.

Real Time PCR was performed in a 20 µl final volume containing 20 ng of cDNA, 1X Power SYBR Green PCR Master Mix (Applied Biosystem, Foster City, CA, USA ), and a mix of forward and reverse primers (1 mmol/l each). The following primers were used for Real Time PCR performed on cDNA: *CYP24A1* forward 5′-TGGCCTACAAGCCCTATATCAC-3′ and reverse 5′-TGCGTAGGAGAAGAACAGGC-3′, *CDKN1A* forward 5′-AGGGGACAGCAGAGGAAG-3′ and reverse 5′-GCGTTTGGAGTGGTAGAAATCTG-3′, *OCN* forward 5′-CACCGAGACACCATGAGAGC-3′ and reverse 5′-CTGCTTGGACACAAAGGCTGC-3′, *TRPV6* forward 5′-GGACAACACCCTCTTACAGCA-3′ and reverse 5′-CCAGCACCATGAAGGCATA-3′, *CaBP9k* forward 5′-TCACTATTGGGCAACCAGACA-3′ and reverse 5′-AGGGTGTTTGGACCTTTGAGT-3′. Human *GAPDH* was used as a housekeeping gene for both Real Time-PCR experiments: forward 5′-TCGACAGTCAGCCGCATCTT-3′ and reverse 5′-AGGCGCCCAATACGACCAAA-3′. Real Time PCR was performed on thermocycler StepOne plus (Applied Biosystems, Foster City, CA, USA) using the following parameters: 95 °C for 10 min followed by 40 cycles of 95° C for 15 s, 60 °C for 30 s, and 72 °C for 30 s. Relative quantification was performed using Delta Delta Ct (2^−ΔΔCt^) method.

### Subjects

Subjects were recruited in a previous study on 117 subjects aged 18–21 who voluntarily agreed to complete a cross-sectional study between October 2017 and December 2018^18^, whose serum samples were frozen at − 80 °C. Here we report the findings on 100 white Caucasian males, 50 from the PFAS exposed area and 50 from the general population. Details on subjects selection are available elsewhere^18^. Briefly, Based on geographical distribution of PFAS pollution from Regional authorities, subjects were grouped on the basis of their residence since birth. subjects who moved in the exposure area after birth were not included in the study. A standardized questionnaire was used to collect information about smoking habits, alcohol misuse, and calcium intake. Included subjects were free from medications known to affect calcium metabolism, such as calcium tablets, bisphosphonate, and corticosteroids, vitamin D supplementation, and testosterone replacement therapy. Risky alcohol consumption was defined as daily alcohol intake at or above 30 g/day. Written informed consent was obtained from all subjects, and the study was approved by the Research Ethics Committee of the University Hospital of Padova (N. 2208 P). The investigation was performed according to the principles of the Declaration of Helsinki. Participants did not receive any reimbursement.

### Biochemical analyses

Hormone serum levels and bone densitometry were analysed as described elsewhere^60^. Briefly, 25-hydroxyvitamin D levels were assessed with direct, competitive chemiluminescent immunoassay (LIAISON 25 OH vitamin D TOTAL Assay, DiaSorin Inc.). PTH serum levels were determined with a direct, two-site, sandwich type chemiluminescent immunoassay (LIAISON N-TACT PTH, DiaSorin Inc. Stillwater, MN). Calcium and phosphate concentrations were measured by QuantiChrom calcium or phosphorus assay kits (BioAssay Systems, Hayward, CA, USA). All determinations were performed according to manufacturer’s instructions.

### PFOA quantification by mass spectrometry

The quantification of perfluorooctanoic acid (PFOA) was processed as previously described^21^ on reversed-phase liquid chromatography coupled with high-resolution mass spectrometry (LC–MS) (Agilent Varian 320; Agilent Technologies, Santa Clara, CA). Briefly, each sample was dissolved in acetonitrile, and fixed amounts of the stable isotope-labelled internal standard were added (MPFOA [marked PFOA]; Wellington Laboratories, Ontario, Canada). Standard mixture was used at increasing concentrations (PFAC-MXB; Wellington Laboratories) together with isotope-labelled internal standard (MPFOA) at fixed concentrations. This solution was analysed by LC–MS. PFOA was identified by comparing the retention time and mass spectra (i.e., m/z value and isotopic pattern). LOQ was 0.2 ng/mL. Quantification was calculated using the corresponding calibration curve.

### Statistical analysis

Statistical analysis of the data was conducted with SPSS 23.0 for Windows (SPSS, Chicago, IL). The results are expressed as means ± SD. Prior to data analysis, the Kolmogorov–Smirnov test was used to check normal distribution of data. Variables not showing normal distribution were log transformed. Levene’s test was used to test the homogeneity of variance among groups. If homogeneity of variance assumption was violated, Welch test was performed and the respective p value was reported. Characteristics of exposed and control subjects were compared by Mann–Whitney U test for continuous variables and by Chi-square test for nominal variables. Analysis of gene expression data was performed by two-way analysis of variance (ANOVA) with post hoc tests for pairwise comparisons. *p* values < 0.05 were considered statistically significant.
